# The von Neumann Entropy for Mixed States

**DOI:** 10.3390/e21010049

**Published:** 2019-01-10

**Authors:** Jorge A. Anaya-Contreras, Héctor M. Moya-Cessa, Arturo Zúñiga-Segundo

**Affiliations:** 1 Instituto Politécnico Nacional, ESFM Departamento de Física, Edificio 9 Unidad Profesional Adolfo López Mateos, 07738 México D.F., Mexico; 2 Instituto Nacional de Astrofísica, Óptica y Electrónica, Calle Luis Enrique Erro No. 1, 72840 Sta. María Tonantzintla, Pue., Mexico

**Keywords:** von Neumann entropy, mixed states, Araki–Lieb inequality, atom-field interaction

## Abstract

The Araki–Lieb inequality is commonly used to calculate the entropy of subsystems when they are initially in pure states, as this forces the entropy of the two subsystems to be equal after the complete system evolves. Then, it is easy to calculate the entropy of a large subsystem by finding the entropy of the small one. To the best of our knowledge, there does not exist a way of calculating the entropy when one of the subsystems is initially in a mixed state. For the case of a two-level atom interacting with a quantized field, we show that it is possible to use the Araki–Lieb inequality and find the von Neumann entropy for the large (infinite) system. We show this in the two-level atom-field interaction.

## 1. Introduction

It is well known that the atomic inversion for a two-level atom interacting with a quantized field suffers collapses and revivals of Rabi oscillations [1,2,3]. The revivals may be considered as an indicator of the nature of the photon distribution of the initial field inside the cavity, because the atomic inversion depends on it:

(1) $$W\left(t\right)=\sum_{n=0}^{\infty }P_{n}\cos\left(2\lambda t\sqrt{n+1}\right),$$

where λ is the atom-field interaction constant and $P_{n}$ is the photon distribution.

For instance, in the case that a squeezed field is considered, the atomic inversion shows so-called ringing revivals that give us information that such a non-classical field was used as an initial field state [4,5]. However, different initial fields may produce the same atomic inversions, namely a coherent state (|α〉) and a statistical mixture of coherent states ($\frac{1}{2}[|\alpha \rangle \langle \alpha |+|-\alpha \rangle \langle -\alpha ]$), which produce the same atomic inversion as both have the same photon distribution:

(2) $$P_{n}=e^{-{\left|\alpha \right|}^{2}}\frac{{\left|\alpha \right|}^{2n}}{n!},$$

with α the amplitude of the coherent state.

Both states that have the same distribution (2) are completely different, as the coherent state is a pure state, while the statistical mixture is a mixed state. On the other hand, decoherence plays a strong role in the purity of the states of quantum systems [6]. For instance, it is well known that a superposition of coherent states (|ψ〉∝|α〉+|−α〉), a so-called Schrödinger cat state, reduces to a statistical mixture of coherent states when subjected to dissipation [7,8,9,10]. The way that coupling parameters are effectively affected by an environment has been already studied on qubit–field interactions [11]. This is an indication that, in order to have better knowledge of a given field, information is also needed about its degree of purity. One of the most important quantities to measure the degree of purity of a state is von Neumann entropy [12], which, together with the atomic inversion, may give information about the initial states used in a given experiment [13,14]. For instance, if the entropy of a quantized field initially in a squeezed state, after some interaction time, is close to zero and the atomic inversion is in the collapse region, it is known that a superposition of squeezed states is a good approximation to the state generated [5].

In summary, the atomic inversion can give us some information about the initial state of the field and, together with entropy, can tell us if the initial field was in a pure state or in a mixed state [15,16].

Because we are studying the interaction of two subsystems, namely atom and field (although it may be generalized to other systems, for instance ion–laser interaction [13]), a quantity of interest is the so-called Araki–Lieb inequality [17]:

(3) $$|S_{A}-S_{B}|\leq S_{AB}\leq S_{A}+S_{B}$$

where $S_{AB}$ is the von Neumann entropy of the total system and $S_{A}$ and $S_{B}$ are the entropies of the subsystems A and B, respectively. This inequality is of great help when one needs to calculate the entropy of a subsystem if the two subsystems are initially in pure states, because the total wavefunction being also in a pure state, it is maintained pure through unitary evolution. In other words, the fact that $S_{AB}=0$ results in both entropies being equal, $S_{A}=S_{B}$, allows us to calculate the entropy of one of the subsystems from the other subsystem entropy. In the case of the (two-level) atom-field interaction, because the atom lives in a two-dimensional Hilbert space, its entropy is easy to calculate, while the entropy for the field (living in an infinite Hilbert space) is complicated [15]. However, the Araki–Lieb inequality tells us that both are the same, provided the initial states for the atom and field were initially in pure states.

A question arises: Is it possible to calculate the entropy of the field when it is initially in a mixed state, namely a mixture of coherent states? Recently, we tried to give an answer to this question, but were only able to deliver a positive answer for certain periods of time, but not for the complete evolution [18]. This is because in such a case, the above triangle inequality seems to be useless, and there is not a general answer.

Phoenix and Knight showed how the field entropy for an initial coherent state can be calculated analytically for initial pure states for the atom and the field without making use of the Araki–Lieb inequality. They were able to find eigenstates and eigenvalues of the field density matrix and, from it, to construct the entropy. Phoenix later applied the same method to compute the entropy for a field subject to decay. Calculations of this type are already complicated for pure initial states (for both subsystems).

In this contribution, we show a possible answer to the problem of finding the field entropy even though any of the subsystems is in a mixed state. In this case, $S_{AB}\neq 0$, and therefore, we cannot say much about the subsystems’ entropies. However, we will introduce the idea of a virtual four-level atom that will allow as to use the Araki–Lieb inequality. The solution we provide may be easily generalized to more complicated interactions (atoms with more levels) or more complicated mixtures of atomic or field states.

We will start by introducing our method, which consists of the fact that, once we trace over the atomic basis in order to obtain a field density matrix, we use the concept of virtual (many-level) atoms, which will be key for our calculation. We finally analyze, as an example, the atom-field interaction in some detail when different initial mixed states are considered, i.e., specifically, when the Araki–Lieb inequality cannot be used to obtain the entropy of the large system (field) in terms of the small one (atom).

## 2. Schmidt Decomposition

The Schmidt decomposition [19] is a useful mathematical tool that plays an important role in one of the key features of quantum mechanics, namely the description of entanglement.

Let us consider a wave function |ψ〉 of an entangled state that describes the interaction between an *n*-level system (an atom, for simplicity) and an infinite-level system (a quantized field):

(4) $$|\psi \rangle =\sum_{k=1}^{n}|\psi_{k}\rangle |a_{k}\rangle ,$$

where ${\left\{|\psi_{k}\rangle \right\}}_{F}$ and ${\left\{|a_{k}\rangle \right\}}_{A}$ are a set of (unnormalized) field and atomic states, respectively, which satisfy, in general, the following conditions:

(5) $$\langle \psi_{i}|\psi_{j}\rangle \neq 0\;\;,\langle a_{i}|a_{j}\rangle =\delta_{ij}\;\;,\forall \;\;\;i,j\in \left\{1,2,\ldots ,n\right\}.$$

We should stress that the above state is not written as a Schmidt decomposition because the states $|\psi_{j}\rangle$ are neither orthogonal, nor normalized.

Schmidt decomposition [19] states that there exist a couple of orthonormal bases ${\left\{|\Psi_{k}\rangle \right\}}_{F}$ and ${\left\{|A_{k}\rangle \right\}}_{A}$ and real, non-negative numbers, $\lambda_{k}$, such that:

(6) $$|\psi \rangle =\sum_{k=1}^{n}\sqrt{\lambda_{k}}|\Psi_{k}\rangle |A_{k}\rangle .$$

The fact may be noted that a density matrix from the state (6) may be handled easily because it is formed by normalized states, and therefore, functions of it may be calculated in a straightforward manner, while the form written in Equation (4) is not easy to handle. This fact will become clearer in the next section.

Moreover, the following quantity:

(7) $$\sum_{k=1}^{n}|\psi_{k}\rangle \langle \psi_{k}|=\sum_{k=1}^{n}\lambda_{k}|\Psi_{k}\rangle \langle \Psi_{k}|$$

is an invariant for such an entangled state.

## 3. Entropy Associated with an *n*-Level System: Mixed States

Consider ${\hat{\rho }}_{M}$ a density operator for a mixed state, defined by:

(8) $${\hat{\rho }}_{M}=\sum_{k=1}^{n}|\psi_{k}\rangle \langle \psi_{k}|,$$

with:

(9) $$\sum_{k=1}^{n}\langle \psi_{k}|\psi_{k}\rangle =1.$$

Because of the invariant Equation (7), it may be rewritten as:

(10) $${\hat{\rho }}_{M}=\sum_{k=1}^{n}\lambda_{k}|\Psi_{k}\rangle \langle \Psi_{k}|,$$

and, as the wavefunctions ${\left\{|\Psi_{k}\rangle \right\}}_{F}$ are orthonormal, the von Neumann entropy (defined as $S=-Tr\{\hat{\rho }\ln\hat{\rho }\}$) may be easily found:

(11) $$S_{M}=-\sum_{k=1}^{n}\lambda_{k}\ln\lambda_{k}.$$

In what follows, we will show that the entropy to the mixed state (field), Equation (10), is equal to the entropy associated with a virtual atom and will verify that this fact is consistent with the Araki–Lieb inequality [17]. In order to achieve this, we consider the density operator for the composed virtual-atom-field pure state, Equation (6),

(12) $$\hat{\rho }=\sum_{j=1}^{n}\sum_{k=1}^{n}\sqrt{\lambda_{j}\lambda_{k}}|\Psi_{j}\rangle |A_{j}\rangle \langle A_{k}|\langle \Psi_{k}|,$$

which, by tracing over the field states, produces the atomic density operator:

(13) $${\hat{\rho }}_{A}=\sum_{k=1}^{n}\lambda_{k}|A_{k}\rangle \langle A_{k}|,$$

from which we can easily obtain the (virtual) atomic entropy:

(14) $$S_{A}=-\sum_{k=1}^{n}\lambda_{k}\ln\lambda_{k}.$$

In addition, if we trace the total density matrix, Equation (12), over the atomic states, we find that the field entropy may be written as:

(15) $$S_{F}=-\sum_{k=1}^{n}\lambda_{k}\ln\lambda_{k},$$

i.e., both entropies are equal, $S_{F}=S_{A}$, and they are also equal to the entropy associated with the mixed state (10). Therefore, in order to find the entropy for a mixed state (8), one may construct an associated virtual atom, then calculate its entropy, and, by virtue of the Araki–Lieb inequality, associate such atomic entropy with the field mixed state. It is possible to use the Araki–Lieb inequality because the density matrix (12) is precisely a density matrix for a pure state, making the total entropy of the composed system equal to zero. Moreover, although both entropies, for the field and virtual atom, are not zero at time t=0, they have the same value, not violating the Araki–Lieb inequality.

It is also important to stress here that the maximum value of $S_{M}$ is lnn, because the virtual atom will be maximally entangled when all the probability amplitudes, $\sqrt{\lambda_{k}}$, for k=1,2,…,n, reach the same value, that is $\lambda_{k}=1/n$.

## 4. Interaction between a Two-Level and a Quantized Field

In order to apply our findings, we use as an example the well-known Jaynes–Cummings model [20], whose interaction Hamiltonian reads:

(16) $${\hat{H}}_{I}=\lambda \left({\hat{a}}^{†}\sigma_{-}+\hat{a}\sigma_{+}\right),$$

which describes the interaction between a two-level atom and a quantized field in the rotating wave approximation. The interaction constant, λ, defines the rate at which the atom and the field exchange energy. The operators $\hat{a}$ and ${\hat{a}}^{†}$ are the field annihilation and creation operators, respectively, while $\sigma_{-}$ and $\sigma_{+}$ are the atomic lowering and raising Pauli operators. It is not difficult to obtain the evolution operator for the Hamiltonian above, which reads [1]:

(17) $${\hat{U}}_{I}=\left(\begin{array}{cc} \cos\left(\lambda t\sqrt{\hat{a}{\hat{a}}^{†}}\right) & -i\;\hat{V}\sin\left(\lambda t\sqrt{{\hat{a}}^{†}\hat{a}}\right)\\ -i\;{\hat{V}}^{†}\sin\left(\lambda t\sqrt{\hat{a}{\hat{a}}^{†}}\right) & \cos\left(\lambda t\sqrt{{\hat{a}}^{†}\hat{a}}\right) \end{array}\right),$$

with $\hat{V}$ and ${\hat{V}}^{†}$ the London phase operators.

### 4.1. Initial Field in a Mixed State and Atom in an Excited State

First, we consider the case of the field initially in a mixture of two coherent states and the atom in its excited state, i.e., $\hat{\rho }\left(0\right)=\left(C|\alpha \rangle \langle \alpha |+(1-C)|\beta \rangle \langle \beta |\right)|e\rangle \langle e|$, for which the evolved density matrix reads:

(18) $$\hat{\rho }=\left(\begin{array}{cc} \left|\psi_{1}\rangle \langle \psi_{1}|+|\psi_{2}\rangle \langle \psi_{2}\right| & \left|\psi_{1}\rangle \langle \psi_{3}|+|\psi_{2}\rangle \langle \psi_{4}\right|\\ \left|\psi_{3}\rangle \langle \psi_{1}|+|\psi_{4}\rangle \langle \psi_{2}\right| & \left|\psi_{3}\rangle \langle \psi_{3}|+|\psi_{4}\rangle \langle \psi_{4}\right| \end{array}\right),$$

with:

(19) $$\begin{array}{ccc} |\psi_{1}\rangle & = & \sqrt{C}\cos\left(\lambda t\sqrt{\hat{n}+1}\right)|\alpha \rangle ,\\ |\psi_{2}\rangle & = & \sqrt{1-C}\cos\left(\lambda t\sqrt{\hat{n}+1}\right)|\beta \rangle ,\\ |\psi_{3}\rangle & = & -i\;\sqrt{C}{\hat{V}}^{†}\sin\left(\lambda t\sqrt{\hat{n}+1}\right)|\alpha \rangle ,\\ |\psi_{4}\rangle & = & -i\;\sqrt{1-C}{\hat{V}}^{†}\sin\left(\lambda t\sqrt{\hat{n}+1}\right)|\beta \rangle . \end{array}$$

We find the reduced atomic and field density operators by tracing over the field:

(20) $${\hat{\rho }}_{A}=\left(\begin{array}{cc} \langle \psi_{1}|\psi_{1}\rangle +\langle \psi_{2}|\psi_{2}\rangle & {\langle \psi_{1}|\psi_{3}\rangle }^{*}+{\langle \psi_{2}|\psi_{4}\rangle }^{*}\\ \langle \psi_{1}|\psi_{3}\rangle +\langle \psi_{2}|\psi_{4}\rangle & \langle \psi_{3}|\psi_{3}\rangle +\langle \psi_{4}|\psi_{4}\rangle \end{array}\right),$$

and the atomic basis:

(21) $${\hat{\rho }}_{F}=|\psi_{1}\rangle \langle \psi_{1}|+|\psi_{2}\rangle \langle \psi_{2}|+|\psi_{3}\rangle \langle \psi_{3}|+|\psi_{4}\rangle \langle \psi_{4}|,$$

respectively.

Note now that Equation (21) is precisely the invariant defined in Equation (7), but for a virtual four-level atom.

Because of Equation (19), the matrix elements of the density operator associated with the virtual four-level system are given by $P_{ij}=\langle \psi_{i}|\psi_{j}\rangle$.

Therefore, the entropy, $S_{F}$, and the purity parameter, $\xi_{F}=1-Tr\left\{{\hat{\rho }}_{F}^{2}\right\}$, may be easily calculated as:

(22) $$S_{F}=-\lambda_{1}\ln\lambda_{1}-\lambda_{2}\ln\lambda_{2}-\lambda_{3}\ln\lambda_{3}-\lambda_{4}\ln\lambda_{4}$$

and:

(23) $$\begin{array}{ccc} \xi_{F} & = & 1-|P_{11}{|}^{2}-|P_{22}{|}^{2}-|P_{33}{|}^{2}-|P_{44}{|}^{2}-2|P_{12}{|}^{2}\\ & & -2|P_{13}{|}^{2}-2|P_{14}{|}^{2}-2|P_{23}{|}^{2}-2|P_{24}{|}^{2}-2|P_{34}{|}^{2}, \end{array}$$

where the $\lambda_{j}$’s are the solutions of the determinant equation:

(24) $$\det\left(\begin{array}{cccc} P_{11}-\lambda & P_{12}^{*} & P_{13}^{*} & P_{14}^{*}\\ P_{12} & P_{22}-\lambda & P_{23}^{*} & P_{24}^{*}\\ P_{13} & P_{23} & P_{33}-\lambda & P_{34}^{*}\\ P_{14} & P_{24} & P_{34} & P_{44}-\lambda \end{array}\right)=0.$$

Of course, the entropy for the real two-level atom is simply described by:

(25) $$S_{A}=-\lambda_{+}\ln\lambda_{+}-\lambda_{-}\ln\lambda_{-}$$

with:

(26) $$\lambda_{\pm }=\frac{1}{2}\pm \frac{1}{2}\sqrt{{\left(P_{11}+P_{22}-P_{33}-P_{44}\right)}^{2}+4|P_{13}+P_{24}{|}^{2}}.$$

**Figure 1:**
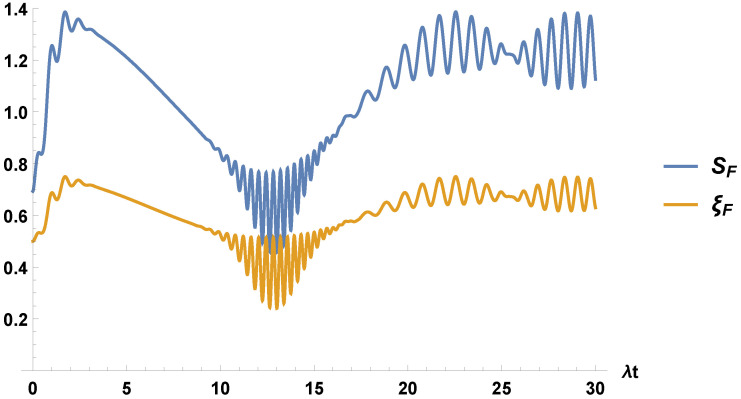
We plot the field entropy, $S_{F}$, and the purity parameter, $\xi_{F}$, as a function of λt for an initial field mixture of coherent states, with α=4, β=−4, C=1/2, and the atom initially in its excited state.

In Figure 1, we plot the entropy, $S_{F}$, and the purity parameter, $\xi_{F}$, which, as should be expected, show the same behavior. We were able to calculate in [18] the field entropy, analytically, only for certain periods of time. The figure presented there coincides with the $S_{F}$ of Figure 1 for several time intervals. As stated before, in [18], we were able calculate correctly the entropy for some time intervals, precisely the ones where they match. Therefore, Figure 1 extends the validity to intervals of time from about λt≈10 to λt≈17. Note that, although we are considering a two-level atom, the maximum of the entropy goes up to ln4, defined by our four-level virtual system.

**Figure 2:**
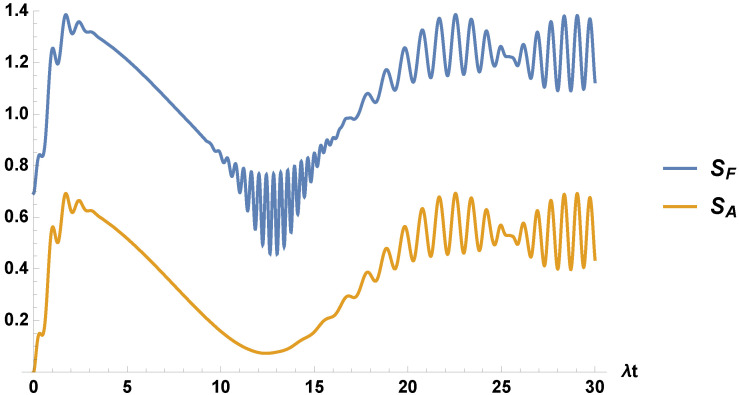
We plot the field entropy, $S_{F}$, and the atomic entropy, $S_{A}$, for the same parameters used in Figure 1.

In Figure 2, we plot the entropies for the field and the two-level atom. They show different behaviors, as should be expected, as they, because the Araki–Lieb inequalities are expected to be different (the entropies of the field and the virtual four-level atom are the same, but differ from the two-level atom). In fact, besides the difference by an amount ln2, the atomic entropy lacks the oscillations present in the field entropy for the period of time from about λt≈10 to λt≈17.

### 4.2. Atom Initially in a Mixture of States and Field in a Coherent State

The formalism to calculate the entropy for mixed states can be extended to the case in which, not the field, but the atom, is in a mixed state. In fact, this may be generalized to even more complicated cases, but we feel that it is enough to present this other case. Consider then an initial atom-field density matrix $\hat{\rho }\left(0\right)=\left(C|e\rangle \langle e|+(1-C)|g\rangle \langle g|\right)|\alpha \rangle \langle \alpha |$. In this case, its evolution is described by:

(27) $$\hat{\rho }=\left(\begin{array}{cc} \left|\phi_{1}\rangle \langle \phi_{1}|+|\phi_{2}\rangle \langle \phi_{2}\right| & \left|\phi_{1}\rangle \langle \phi_{3}|+|\phi_{2}\rangle \langle \phi_{4}\right|\\ \left|\phi_{3}\rangle \langle \phi_{1}|+|\phi_{4}\rangle \langle \phi_{2}\right| & \left|\phi_{3}\rangle \langle \phi_{3}|+|\phi_{4}\rangle \langle \phi_{4}\right| \end{array}\right),$$

where:

(28) $$\begin{array}{ccc} |\phi_{1}\rangle & = & \sqrt{C}\cos\left(\lambda t\sqrt{\hat{n}+1}\right)|\alpha \rangle ,\\ |\phi_{2}\rangle & = & -i\;\sqrt{1-C}\;\hat{V}\sin\left(\lambda t\sqrt{\hat{n}}\right)|\alpha \rangle ,\\ |\phi_{3}\rangle & = & -i\;\sqrt{C}\;{\hat{V}}^{†}\sin\left(\lambda t\sqrt{\hat{n}+1}\right)|\alpha \rangle ,\\ |\phi_{4}\rangle & = & \sqrt{1-C}\cos\left(\lambda t\sqrt{\hat{n}}\right)|\alpha \rangle . \end{array}$$

**Figure 3:**
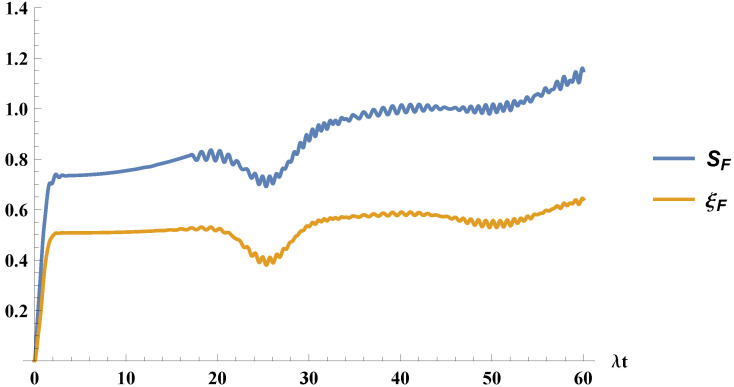
We plot the entropy, $S_{F}$, and the field purity parameter, $\xi_{F}$, for the atom initially in a mixture of ground and excited states, with C=1/2 and the field initially in a coherent state with α=4.

The atomic and field reduced density operators read:

(29) $${\hat{\rho }}_{A}=\left(\begin{array}{cc} \langle \phi_{1}|\phi_{1}\rangle +\langle \phi_{2}|\phi_{2}\rangle & {\langle \phi_{1}|\phi_{3}\rangle }^{*}+{\langle \phi_{2}|\phi_{4}\rangle }^{*}\\ \langle \phi_{1}|\phi_{3}\rangle +\langle \phi_{2}|\phi_{4}\rangle & \langle \phi_{3}|\phi_{3}\rangle +\langle \phi_{4}|\phi_{4}\rangle \end{array}\right),$$

and:

(30) $${\hat{\rho }}_{F}=|\phi_{1}\rangle \langle \phi_{1}|+|\phi_{2}\rangle \langle \phi_{2}|+|\phi_{3}\rangle \langle \phi_{3}|+|\phi_{4}\rangle \langle \phi_{4}|,$$

respectively. In a similar fashion as the case previously described, we note that Equation (30) is nothing but the invariant defined in Equation (7), again for a four-level virtual atom. We can follow the procedure described above and plot in Figure 3 the field entropy, $S_{F}$, and field purity parameter, $\xi_{F}$, to show they again, as expected, have the same behavior.

**Figure 4:**
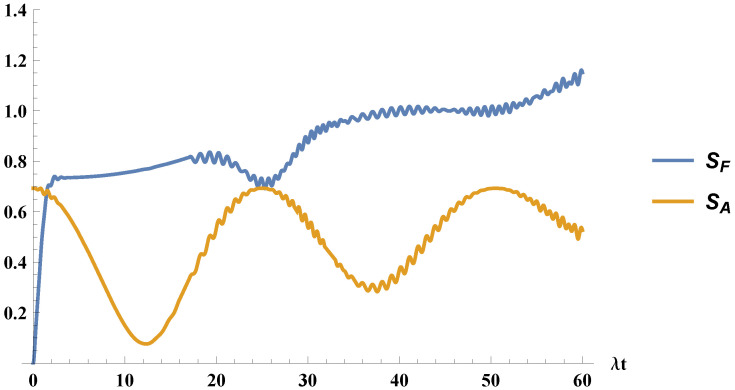
We plot the time evolution of the entropies, for the field, $S_{F}$, and for the atom, $S_{A}$, for the atom initially in an statistical mixture of excited and ground states, with C=1/2 and the field initially in a coherent state with α=4.

Finally, we show in Figure 4 the field and atomic entropies. In this case, they show a completely different behavior, unlike Figure 2.

## 5. Conclusions

We have shown that in the atom-field interaction, although the atom or the field may be initially in mixed states, it is possible, by using the Araki–Lieb inequality and the concept of virtual (extended) atoms, to calculate the entropy of the field. Although the small system (in this case, the two-level atom) continues to deliver the information of the big system (the field), its Hilbert space should be extended, in fact doubled, for us to be able to extract information about the complete system. To be clearer, if we consider the density matrix a field in the form:

(31) $$\rho_{F}=\frac{1}{N}\sum_{k=0}^{N-1}|\alpha_{k}\rangle \langle \alpha_{k}|$$

we need to consider a virtual *N*-level atom for which the eigenvalues are properly calculated in order to find the entropy for the above mixed density matrix.
